# Assessment of the relationship between the maxillary sinus and the canine root tip using cone beam computed tomography

**DOI:** 10.1186/s12903-021-01700-2

**Published:** 2021-07-10

**Authors:** Leila Khojastepour, Najmeh Movahhedian, Mohadeseh Zolghadrpour, Mohammad Mahjoori-Ghasrodashti

**Affiliations:** 1 Department of Oral and Maxillofacial Radiology, School of Dentistry, Shiraz University of Medical Sciences, Qom Abad, Ghasrodasht St., Shiraz, Iran; 2 Student Research Committee, School of Dentistry, Shiraz University of Medical Sciences, Shiraz, Iran; 3 Eastman Institute for Oral Health, Rochester, USA

**Keywords:** Maxillary sinus, Anterior extension, Canine apex, Incisor, Cone beam computed tomography

## Abstract

**Background:**

The purpose of the present study is to investigate the relationship between the maxillary sinus and the canine root apices in cone beam computed tomographic images (CBCT) and to assess the amount of extension of the maxillary sinus to the anterior region of the jaw in different sexes and age groups.

**Methods:**

CBCT of 300 individuals (154 males and 146 females) over 20 years (with a mean age of 35.12 ± 8.40 years) were evaluated. The subjects were categorized into three age groups (20–30, 30–40, and 40–50 years). When the maxillary sinus extended to the canine area, the vertical distance between them was measured, and their relationship was classified into three types: I (more than 2 mm distance), II (less than 2 mm distance or in-contact), and III (interlock).

**Results:**

413 out of 600 maxillary sinuses (68.8%) were extended into the canine area or beyond. Among them, 15 maxillary sinuses pneumatized into the incisor area (2.5%). The prevalence of the maxillary sinuses extended to the anterior region of the jaw was not significantly different between genders. However, it was significantly less frequent in the older age group and more frequent on the left side. In addition, the mean amount of anterior extension of the maxillary sinus (mm) was significantly lower in the older age group. Type I was the most frequent vertical relationship between the maxillary sinuses and canine apices with no significant difference in gender, side, and age groups.

**Conclusions:**

In most cases, the maxillary sinus extended to the canine area and sometimes reached the incisor region. This necessitates paying more attention to the maxillary anterior sextant during surgical procedures.

## Background

The maxillary sinuses are the most extensive paranasal sinuses, which are very small at birth but expand by physiologic pneumatization until completion of skeletal development [1] or around 20 years [2]. The maxillary sinus often extends from the distal aspect of the canine tooth to the posterior wall of the maxilla above the tuberosity [3]. Occasionally, general practitioners misinterpret the pneumatization of the anterior part of the maxilla by the maxillary sinus as a cystic lesion on intraoral or panoramic radiographs [4]. Evaluation of the relationship between the maxillary sinus and the dentition is essential for dental procedures, such as implant or apical surgeries and orthodontic treatments [5, 6]. Moreover, assessing these anatomical structures is also important since they provide a pathway for infection to spread from the periodontal or peri-apical lesions into maxillary sinuses, which can cause maxillary sinusitis [7–9].

Two- or three-dimensional (2D or 3D) imaging has been used for evaluating paranasal sinuses. However, in cases with accompanying signs and symptoms regarding sinuses or the need for a thorough examination of sinuses prior to surgeries, 3D imagings are the modalities of choice [9]. In this regard, cone-beam computed tomography (CBCT) provides accurate and distortion-free images of the craniofacial bones with lower absorbed radiation dose compared with multi-slice computed tomography [10–12].

Several studies evaluated the relationships between the posterior teeth and the maxillary sinus [4, 13–19], while the anterior part of the maxilla is often assumed as a relatively safe region for surgical interventions. Based on a recent CBCT study [4], the maxillary sinus extends to the canine area in 68.9% of cases and the incisor region in 15.5%. The high frequency of the sinus extension to the canine tooth area necessitates a thorough evaluation of the region regarding the relationship between the maxillary sinus and the dentition. However, so far, there has been only one CBCT study considering the relationship between the canine and the maxillary sinus [20]. Oishi et al. [20] evaluated the proximity of the canine and posterior teeth to the maxillary sinus floor in the standard coronal and sagittal planes and reported significant differences between the measurements in these two planes. This difference was greater for the canine teeth. This can be due to the curved anatomy of the maxilla and the unique position of the canine tooth in the jaw. It seems that corrected planes parallel and perpendicular to the tooth long axis are necessary for more accurate and reliable results regarding the relationship between the canine tooth and the adjacent structures.

The present study aimed to assess the location of the anterior border of the maxillary sinus concerning teeth and the amount of anterior extension of the maxillary sinus (AEMS) beyond the canine tooth long axis and also the vertical distance between the maxillary sinus floor and the canine apices in different sexes and age groups using corrected CBCT planes.

## Methods

This cross-sectional study has been performed following the Declaration of Helsinki and was approved by the Human Ethics Review Committee of the Faculty of Dentistry, University of Medical Sciences, Shiraz, Iran (# IR Sums.Dental.REC.1399.020).

1430 CBCT scans of patients who attended the Oral and Maxillofacial Department of Shiraz Dental School (from May 2017 to May 2020) were reviewed retrospectively. CBCT images were taken for different purposes other than the present study. Written informed consent was obtained from all the patients at the time of radiographic examination for the possible use of their anonymous information in future researches, which may be published. Personal data of all individuals was kept undisclosed. In the end, 300 scans (154 men and 146 women with mean age of 35.12 ± 8.40 years) met the study criteria.

To be included in the study, the field of view of the CBCT images should cover the entire maxilla of individuals over 20 years, and both maxillary canines should completely erupt and develop. Exclusion criteria were distorted CBCT images due to metallic or motion artifacts, history of previous apical surgery, evidence of root resorption/fracture, intra-bony pathologies, supernumerary/missing/extracted or impacted teeth in the maxilla, congenital anomalies, or severe jaw deformities. In addition, high-buccal canines, not being in line with the incisal/occlusal surface of the dentition, were excluded from the study.

All CBCT images were obtained using the New Tom Evo CBCT unit (QR S.R.L. Company, Verona, Italy) with the following technical parameters: 3 mA, 1.8 exposure time, 110 Kvp, 0.3 mm voxel size, axial pitch, and axial thickness of 0.3 mm. The Frankfort horizontal plane of all the subjects was parallel to the floor when acquiring the images. All the measurements were done using NNT software (NNT 9.2 Image Works, Verona, Italy) by two oral and maxillofacial radiologists with consensus. The same observers re-evaluated One-third of CBCT scans (100) after a two-week interval.

**Fig. 1:**
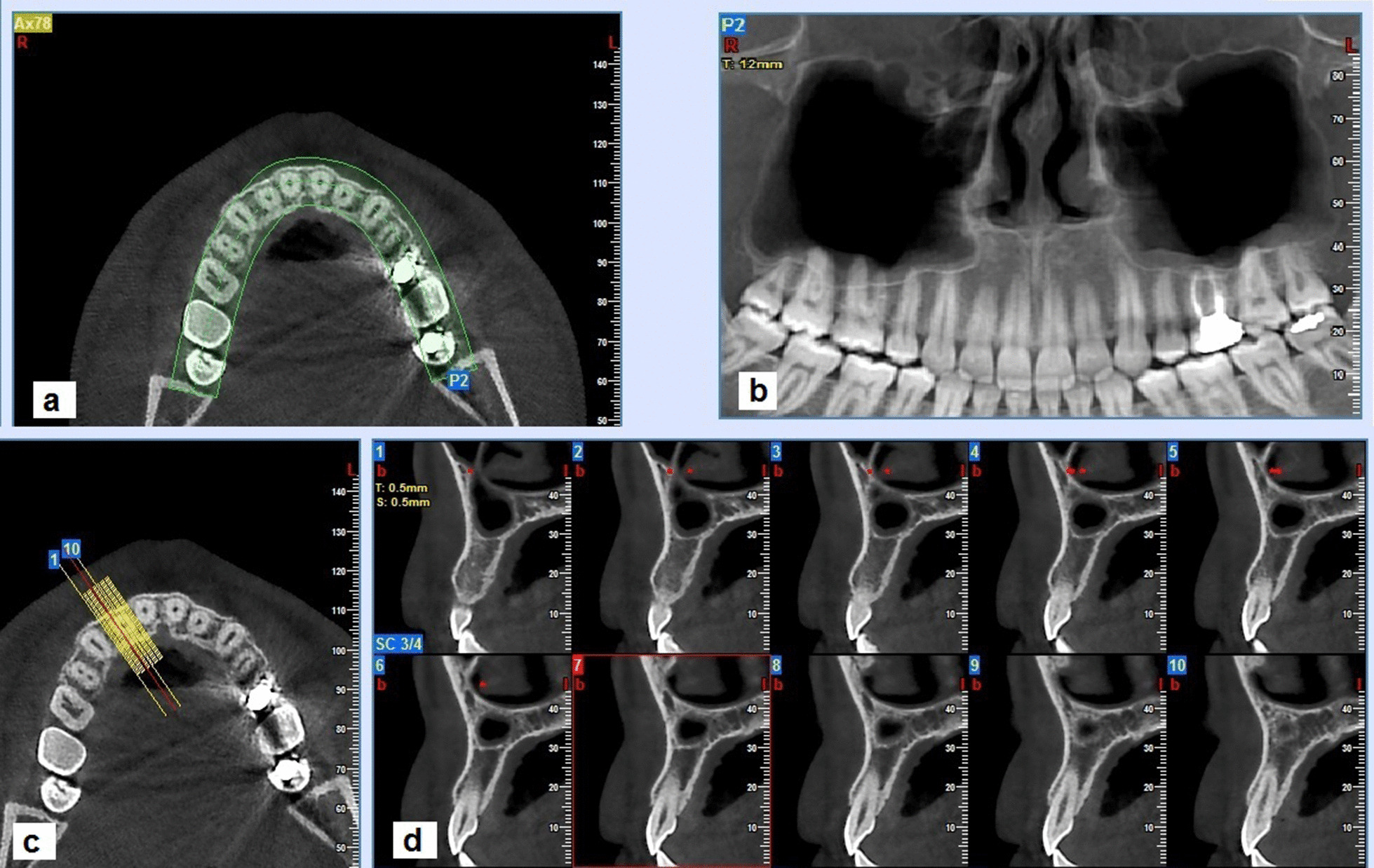
**a** Axial image and curved line which used for panoramic reconstruction. **b** Reconstructed panoramic image. **c** Reference axial image for preparing buccolingual cross-sections. **d** Buccolingual cross-sections perpendicular to dental arch with 0.5 mm thickness and interval in a case of maxillary sinus pneumatization into the lateral incisor area

The study sample was categorized into three age groups: 20–30, 30–40, and 40–50. The subjects were distributed evenly in these age groups as each category of age included 100 scans (200 maxillary sinuses). The distribution of the study subjects in these age groups is presented in Table 1. The evaluators marked the most anterior limit of the anterior border of the maxillary sinus on the axial image for each subject. Then panoramic view was reconstructed based on the curved line drawn parallel to the dental arch at the cervical level of the dentition on the axial image. The axial and reformatted panoramic views served as the reference image for localizing the anterior limit of the maxillary sinus in the cross-sectional images. Bucco-lingual cross-sections were prepared perpendicular to the dental arch with 0.5 mm thickness and interval (Fig. 1). Then we recorded the location of the anterior border of the maxillary sinus in relation to different teeth. In cases with maxillary sinus extension to the canine region, the following evaluations were done: (1) AEMS beyond the canine tooth long axis; (2) the vertical relationship between the maxillary sinus floor and canine apices, and (3) the absolute vertical distance between the maxillary sinus floor and floor of the nasal fossa (MS-NF).

**Table 1:** Comparison of the amount of maxillary sinus extension between different sexes, sides and age group

	Extension of maxillary sinus related to the canine long axis		Total N (%)	*p* Value*
	Anterior N (%)	Posterior N (%)		
*Sex*				
Male	207 (67.21)	101 (32.79)	308 (100)	0.377
Female	206 (70.55)	86 (29.45)	292 (100)	
*Side*				
Right	191 (63.67)	109 (36.33)	300 (100)	0.006
Left	222 (74.00)	78 (26.00)	300 (100)	
*Age group*				
20–30	149 (74.50)	51 (25.50)	200 (100)	0.001
30–40	150 (75.00)	50 (25.00)	200 (100)	
40–50	114 (57.00)	86 (43.00)	200 (100)	

N: Number, %: Percent

**Fig. 2:**
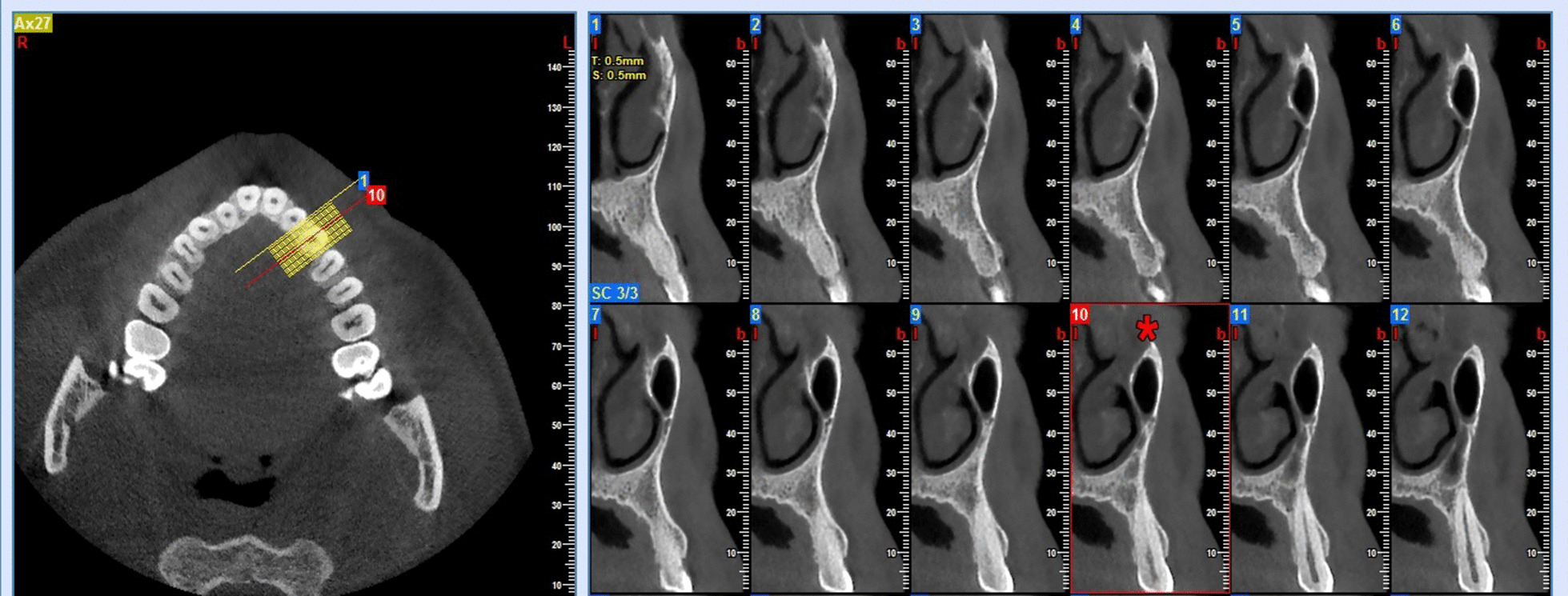
A case with the maxillary sinus extended to the canine area. A star has marked the cross-section passing through the tooth-long axis. In this case, the maxillary sinus can be detected in eight cross-sections beyond the canine tooth long axis. So, the AEMS, in this case, is equal to 4 mm (8 × 0.5)

The number of slices (cross-sections) with sinus pneumatization beyond the canine tooth long axis were counted and multiplied by the slice thickness (0.5 mm) to calculate AEMS beyond the canine tooth long axis (AEMS = Number of slices × Slice thickness). For example, if maxillary sinus pneumatization is noticed on eight slices beyond the canine tooth long axis, the AEMS would be 4 mm (8 × 0.5) (Fig. 2).

The canine tooth long axis was defined as the line passing through the center of the tooth connecting the middle point of the incisal edge to the apex.

**Fig. 3:**
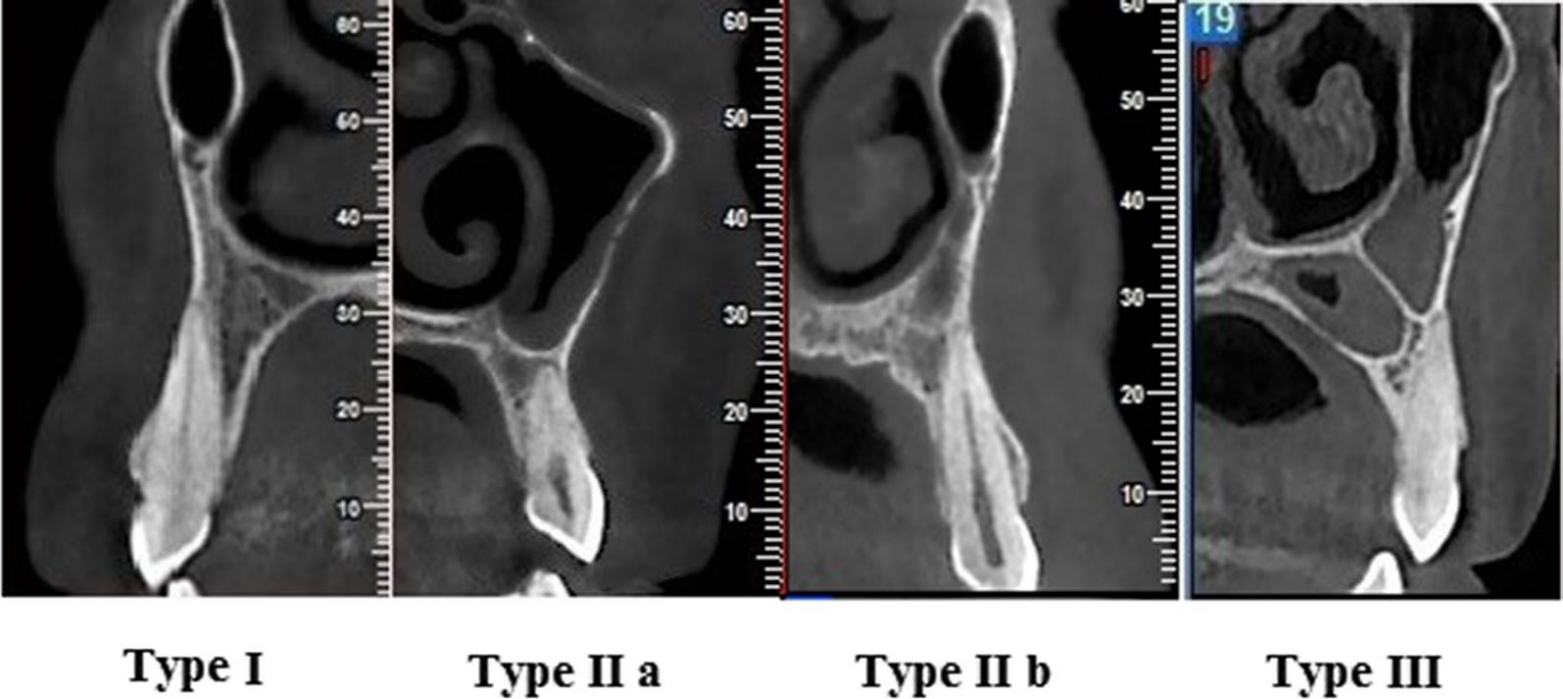
Three types of the vertical relationship between the maxillary sinus floor and canine apex on buccolingual CBCT cross-sections: Type I (more than 2 mm distance), II a (less than 2 mm distance), II b (in contact), and III (interlock) relationship

The vertical relationship between the maxillary sinus floor and canine teeth apices was assessed based on the following classification (Fig. 3):

- *Type I* Apex located below the sinus floor with more than 2 mm distance
- *Type II* Apex located below the sinus floor with less than 2 mm distance (Type II a) or was in contact (Type II b).
- *Type III* Apex located above the sinus floor

**Fig. 4:**
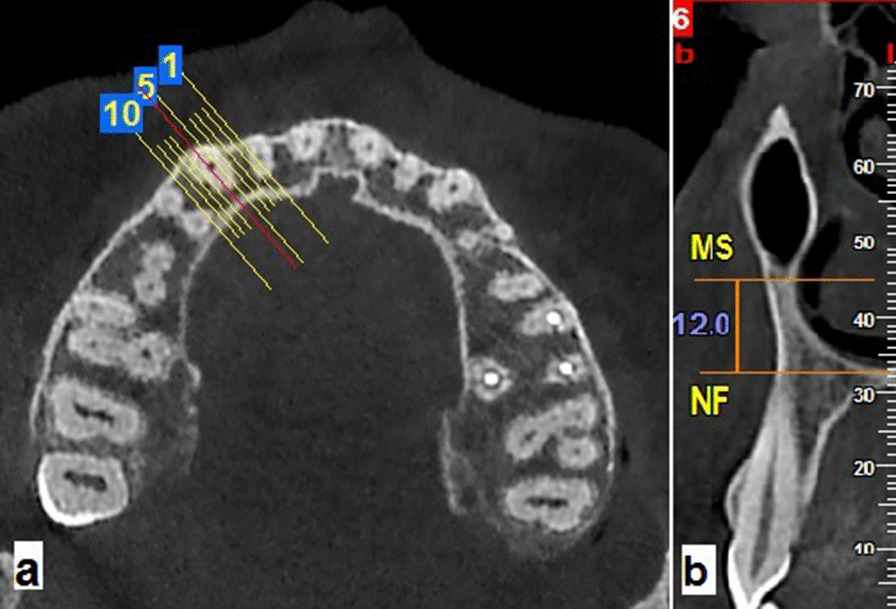
**a** axial reference image and **b** buccolingual cross-section for measurement of the distance between floors of the maxillary sinus (MS) and nasal fossa (NF) at canine tooth long axis

Figure 4 depicts how the vertical distance between the maxillary sinus floor and the nasal floor was measured.

### Statistical analyses

Statistical analyses were conducted using the SPSS software (version 20; IMB; Chicago; IL). Quantitative and qualitative variables were described by mean ± standard deviation (SD) and frequency (percent). We used the Chi-square test to compare the AEMS and the vertical relationship between the maxillary sinus floor and canine apex in different sexes, sides, and age groups. ANOVA test was performed to compare the mean distances (mm) of MS-NF and the anterior extension of the maxillary sinus in different age groups. *p* value < 0.05 was considered statistically significant. The intra-class correlation coefficient (ICC) was also used to evaluate the intra-examiner error.

## Results

CBCT images of 300 individuals (600 maxillary sinuses (met the study inclusion criteria and were evaluated. The mean age of subjects was 35.12 ± 8.40 years. The study sample consisted of 146 (48.7%) women with a mean age of 34.66 ± 8.78 years and 154 (51.3%) men with a mean age of 35.56 ± 8.03 years old. ICC was 96% which is considered a perfect agreement.

Out of 600 maxillary sinuses, 413 (68.83%) extended into the canine area or beyond. 15 sinuses (2.5%), belonged to 8 subjects, involved the incisor region. The location of the anterior border of maxillary sinuses in relation to other teeth was as follows: 149 (24.83%) into the first premolar, 36 (6%) into the second premolar, and 2 (0.33%) into the first molar area.

Sinus extension had bilateral symmetry in 244 individuals (81.33%) and was non-symmetrical in 56 (18.66%). Among cases with symmetric sinus extension, in 178 patients (59.33%), the maxillary sinus extended into the canine region, and in 66 patients (22%), it was located posterior to the canine area bilaterally.

Table 1 shows the maxillary sinus extension related to the canine tooth long axis in different sexes, sides, and age groups. Extension beyond the canine tooth long axis was significantly more frequent on the left side (*p* value = 0.006) and less frequent in the older age group (40–50 years old) (*p* value = 0.001). No sex difference was found in the prevalence of extension of the maxillary sinus to the anterior region of the jaw (*p* value = 0.377).

Regarding the vertical relation between the sinus floor and canine apices, type I was the most frequent. 351 out of 413 (84.99%) maxillary sinuses extended to the canine area had type I vertical relation with canine apices. Type II and III were noted in 37(8.96%) and 25 (6.05%) of such maxillary sinuses, respectively. As shown in Table 2, there were no significant differences between vertical relation of the maxillary sinus floor and canine apices in different sexes, sides, and age groups (*p* value = 0.153, 0.355, and 0.111, respectively).

**Table 2:** Comparison of maxillary sinus and canine apex vertical relationship considering sex, side and age groups

Variable	Maxillary sinus and canine apex relation			Total	p Value*
	Type I N (%)	Type II N (%)	Type III N (%)		
*Sex*					
Male	169 (81.64)	23 (11.11)	15 (7.25)	207 (100)	0.153
Female	182 (88.35)	14 (6.79)	10 (4.85)	206 (100)	
*Side*					
Right	160 (83.77)	16 (8.37)	15 (7.85)	191 (100)	0.355
Left	191 (86.03)	21 (9.50)	10 (4.52)	222 (100)	
*Age group*					
20–30	124 (83.22)	15 (10.07)	10 (6.71)	149 (100)	0.111
30–40	122 (81.33)	15 (10.00)	13 (8.67)	150 (100)	
40–50	105 (92.11)	7 (6.14)	2 (1.75)	114 (100)	

N: Number, %: Percent

In cases with extended maxillary sinus into the canine region (type I and II), the mean vertical distance between the maxillary sinus floor and canine apex was 11.99 ± 5.97 mm. 25 canine apices were located above the maxillary sinus floor (Type III). Their distances to the maxillary sinus floor were evaluated separately. The mean distance in this group was 4.71 ± 3.83 mm. The distance between the maxillary sinus floor and canine apex was not statistically different between age groups (*p* value = 0.207). There was no significant difference between the right and left sides in this regard.

In most cases with extended maxillary sinuses to the canine area, the nasal floor was located below the sinus floor. The mean absolute distance of MS-NF was 10.13 ± 4.76 mm in the study population, 9.84 ± 4.77 mm for the right, and 9.49 ± 4.60 mm for the left side. The difference between these values was not statistically significant.

**Fig. 5:**
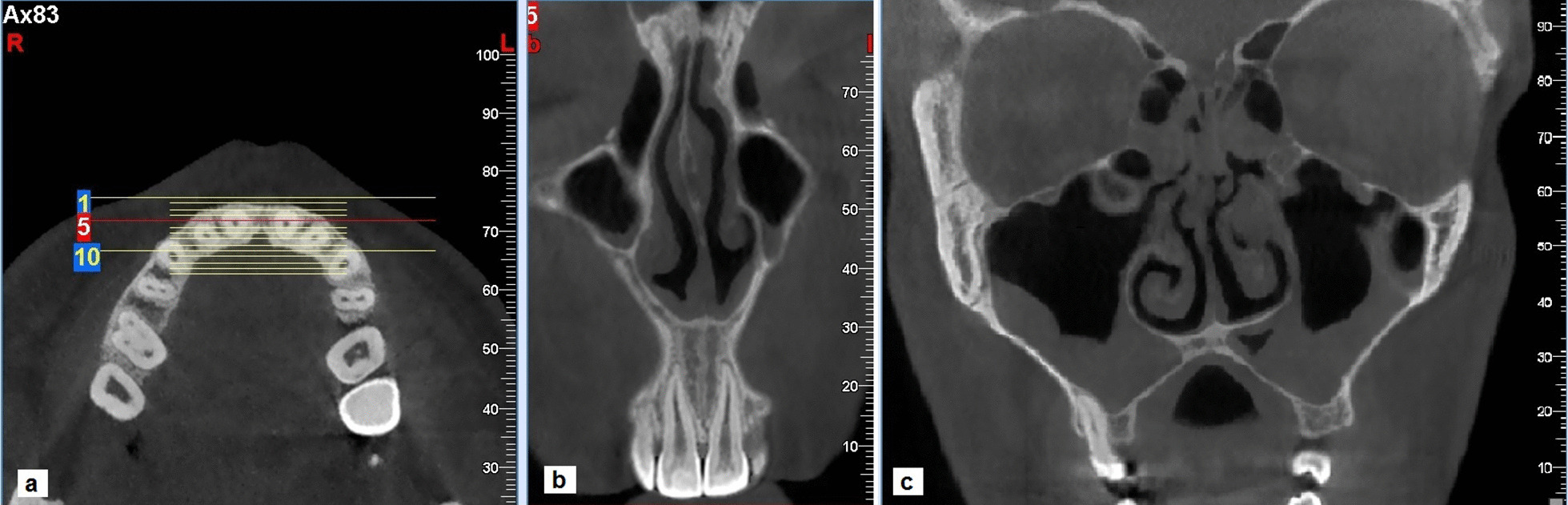
A case of extensive maxillary sinus pneumatization: **a** reference axial image **b** the extension to the central incisor region. **c** involvement of the entire hard palate

The mean of AEMS beyond the maxillary canine long axis was 2.03 ± 1.17 mm with a maximum extension of 16 mm. This value was 2.25 ± 1.18 mm for the left side which was significantly more than the right side (2.04 ± 1.08 mm). Extensive pneumatization of the maxillary sinus is detected in one case, which involved the entire hard palate and extended to the central incisor region (Fig. 5).

Table 3 shows that the mean AEMS was significantly lower in the 40–50 years age group compared to the other age groups (*p* value = 0.001). Moreover, the mean distance of MS-NF was significantly higher in the same age group than the two others (*p* value = 0.001).

**Table 3:** Comparison of the anterior extension of maxillary sinus beyond canine tooth and the distances between the maxillary sinus and nasal floors between age groups

Age group	AEMS Mean ± SD (mm)	MS-NF Mean ± SD (mm)
20–30	2.23 ± 1.147^a^	9.24 ± 4.57^a^
30–40	2.19 ± 1.189^a^	10.04 ± 4.93^a^
40–50	1.54 ± 1.044^b^	11.43 ± 4.53^b^
p Value*	0.001	0.001

*AEMS* anterior extension of the maxillary sinus, *MS-NF* distance between maxillary sinus and nasal floor

*Mean values with at least one same letter in superscript were not statistically different

## Discussion

In the present study, we evaluated the amount of anterior extension of maxillary sinus beyond the long axis of the maxillary canine and the relationship between the root apex of the permanent maxillary canine and the maxillary sinus floor in 300 CBCT images.

The proximity of the sinus floor and root apices of the maxillary teeth is critical in several clinical procedures such as endodontic surgery, orthodontic treatment, and implant placement. CBCT scans, with dedicated 3D software, allow image reconstruction in three orthogonal planes and precise morphometric measurements, which provide accurate information for case selection, treatment planning, and avoiding collateral damage during surgery [10–12].

The result of the present study shows that most of the maxillary sinuses (68.83%) extended to the canine area, which is almost equal to Zhang et al. finding (68.9%) [4]. In contrast, Kim et al. [21] and Kopecka et al. [22] reported much lower percentages of canine area pneumatization by maxillary sinus, 33%, and 2.4%, respectively. Regarding the pneumatization of the incisor region, Zhang et al. [4] reported a frequency of 15.5% (12.1% in lateral and 3.4% in central incisor) in their study population. In the present study, however, the anterior border of the maxillary sinus reached the incisor area in only 2.5% of sinuses. The difference in the percentages reported by the studies may be due to ethnicity and the differences in methodology, including the approach used for localizing the maxillary sinus and the number of subjects.

In this study, most of the maxillary sinuses (81.33%) had bilateral symmetry in the location of the anterior border in the jaw. This finding was in accordance with previous studies [2, 23, 24]. Comparably, Shahbazian et al. [25] reported the symmetric morphology of maxillary sinus in 83% of their cases, and according to Hamdy et al. [26], the average linear craniocaudal, anteroposterior, and mediolateral measurements were almost bilaterally matched in all cases. Based on our results, bilateral symmetry was even more common in those maxillary sinuses which extended into the canine area. Further studies are required to confirm this result.

The frequency of maxillary sinus extended to the anterior region was similar in both sexes in the present study. No study evaluated this relationship previously.

The frequency of maxillary sinus extended to the anterior region, and the mean AEMS beyond the canine long axis was significantly lower in older cases (40–50 years) compared with the other age groups. Additionally, in the vertical dimension, the MS-NF was significantly higher in the older age group. These findings confirm those of Belgin et al. [23], Takahashi et al. [27] and Velasco-Torres et al. [28] who found decreasing maxillary sinus volume with increasing age. Jun et al. [1] also showed that the maxillary sinus increases in size until the completion of skeletal development in both sexes. Then, an age-related decrease occurs in its volume. Similarly, Ariji et al. [2] reported increasing maxillary sinus volume up to 20 years of age and a declining process subsequently. Contrary to all these findings, Sahlstrand-Johnson et al. [29] reported that maxillary sinus volume was not related to age in their study sample.

Oishi et al. [20] found a significant negative correlation between age and the distance of the maxillary sinus floor to the root apices of all teeth (posterior teeth and canine) in CBCT images. The same result was found by AL Qasab et al. [30] regarding the distance between the canine apex and maxillary sinus floor in periapical radiographs. In the present study, the vertical distance between the maxillary sinus floor and canine apex was not statistically related to age. Further research with a larger sample size is needed to clarify these conflicting results.

As part of this study, it is found that when the maxillary sinus extended to the canine area, the apices were most commonly (84.99%) located below the sinus floor and at more than 2 mm distance (type I). It was followed by types II and III relationship with much less percentage (8.96% and 6.05% respectively). This order was in accordance with a recent study by Oishi et al. [20]. They defined type 0 for those cases in which the sinus floor did not appear above the root apices. Type I, II, and III are reserved for separate, in-contact, and interlock relationships between the maxillary sinus floor and canine apices. They reported that whenever maxillary sinus appeared above the canine apices, type I was the most, and type III was the least frequent relationship, similar to our findings.

Oishi et al. [20] assessed the proximity of the posterior teeth and the canine tooth to the maxillary sinus floor in standard sagittal and coronal planes and did not consider the anatomic curvature of the jaw, which is especially important in the canine area. In contrast, in the present study, the classification and measurements were done based on bucco-lingual cross-sections perpendicular to the dental arch of each side of the jaw. This corrected plane seems to be more reliable for evaluating the relationship and distance of the root apices and the sinus floor. In the study of Oishi et al. [20], the subjects was not evenly distributed in according to age and most of their sample (184 subjects) were between 18–29 years. Moreover, their sample size weighted toward the female group (225), which was three times more than the male group (76). To minimize the possible biases and to have more accurate results, we tried to have an even subject distribution in different age groups (each age group contained 100 subjects/200 maxillary sinuses) as well as having similar numbers of male (154) and female (146) subjects. However, based on the limited number of older individuals meeting the study's inclusion criteria, we set the upper age at 50 years old. We encourage future studies with a broader age span to overcome this limitation.

Another limitation of the present study is that the effect of facial biotypes has not been considered in the design of the present study. Okşayan et al. [31] reported lower values in maxillary sinus length and width in high-angle subjects. Costea et al. [32] found that the relationship between the maxillary second molar root apices and the maxillary sinus floor is affected by the facial biotype. Future studies can address whether the different facial biotypes influence the canine tooth and maxillary sinus relationship.

## Conclusions

Within the limitations of this study, the following conclusions may be drawn:

1. Most of the maxillary sinuses extended to the canine area
2. In most cases with the maxillary sinus extended to the canine area, the canine apex was located more than 2 mm below of maxillary sinus floor
3. The maxillary sinus could extend into the incisor region. This extension is of particular importance for surgical procedures and implant treatment in the maxillary anterior region
4. Extension of the maxillary sinus to the anterior part of the maxilla was less frequent in the older age group compared to the younger subjects
5. The mean absolute distance between the maxillary sinus and nasal floor was higher in the older age group

## Data Availability

The datasets used and analyzed during the current study are available from the corresponding author on reasonable request.

## Abbreviations

CBCT: Cone-beam computed tomography
2D or 3D: Two- or three-dimensional
AEMS: Anterior extension of the maxillary sinus
MS-NF: Vertical distance between maxillary sinus floor and floor of the nasal fossa
SD: Standard deviation
ICC: Intra-class correlation coefficient
